# P-727. Is there increased risk of RSV infection following SARS-CoV-2 infection in children? An EHR-based cohort study from the RECOVER Program

**DOI:** 10.1093/ofid/ofae631.923

**Published:** 2025-01-29

**Authors:** Suchitra Rao, Andrea J Allen, Mitchell Maltenfort, Asuncion Mejias, Eneida Mendonca, Benjamin Horne, Emily Taylor, Abu Mosa, Anisha Sekar, Vitaly Lorman

**Affiliations:** University of Colorado School of Medicine, Aurora, CO; Children's Hospital of Philadelphia, Philadelphia, Pennsylvania; Children's Hospital of Philadelphia, Philadelphia, Pennsylvania; St Jude Children's Research Hospital, Memphis, TN; Cincinnati Children Childrens Medical Center, Cincinnati, Ohio; Intermountain Health, Salt Lake City, Utah; NYU Grossman School of Medicine, New Tork, New York; University of Missori School of Medicine, Columbia, Missouri; NYU Grossman School of Medicine, New Tork, New York; Children’s Hospital of Philadelphia, Philadelphia, Pennsylvania

## Abstract

**Background:**

Pediatric populations experienced an unprecedented burden of respiratory infections in 2022, raising a hypothesis that SARS-CoV-2 infection could increase the susceptibility to subsequent respiratory infections. Our objectives were to compare the risk of RSV infection among children with and without a prior documented SARS-CoV-2 infection.

Density landscapes of index infections for the comparison cohorts from March 1, 2022 to July 1, 2022, along with density landscapes of the outcome (RSV infection), for the cohort.

**Figure:**
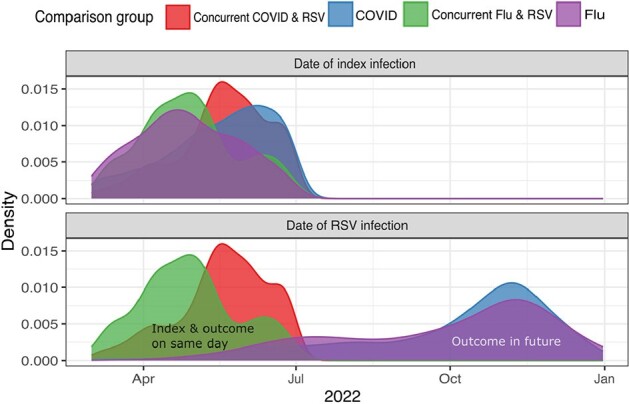

Red shading indicates children with SARS-CoV-2 infection on the same day as their first documented RSV infection, and green shading indicates children with Influenza and RSV on the same day. Purple and blue show the density of influenza and SARS-CoV-2 index cases and subsequent RSV infections not occurring on the same day, respectively.

**Methods:**

This retrospective cohort study utilized inpatient and outpatient Electronic Health Record (EHR) data from 40 PCORnet sites in the US of children < 5 years of age with SARS-CoV-2 infection (test or coded) between March 1, 2022 and July 1, 2022. Comparison groups included a) children with influenza infection (PCR confirmed or coded) and b) children with a respiratory infection and no evidence of SARS-CoV-2 or influenza infection. The primary outcome of interest was RSV infection in the subsequent 15-180 days (PCR-confirmed or coded). Inverse probability of treatment weighting (IPTW) controlled for factors associated with the difference in exposures, including race, ethnicity, age, and date of infection. A weighted logistic regression model produced odds ratios for RSV infection via doubly robust analyses, with sensitivity analyses accounting for different outcome windows.

Odds ratios of weighted, adjusted, and completely unweighted or adjusted models evaluating RSV infection in the 3 windows of follow up (0-60 days, 1-60 days, and 15-180 days) following SARS-CoV-2 infection compared with RSV infection following influenza infection.

**Figure:**
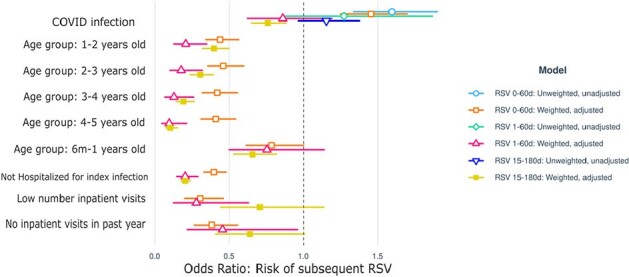

Square and triangle shapes represent odds ratios, lines represent 95% confidence intervals. Each color represents a different model with varied time period of evaluation, weighting and adjustment.

**Results:**

The cohort consisted of 21,537 children with SARS-CoV-2 infection, 8,317 with influenza infection, and 59,798 with another acute respiratory infection. Distributions of index and RSV infection dates are shown in Figure 1. In weighted analyses, we found a 30% higher risk of RSV infection in the 0-60 days following SARS-CoV-2 infection; however, this increase was driven by co-infections (of the 3.06% of SARS-CoV-2 patients with RSV in the subsequent 0-60 days, two thirds had RSV/SARS-CoV-2 coinfection). When assessing the evaluation period of 1-60 days or 15-180 days, we did not find a significant difference in the risk of RSV infection (Figure 2).

**Conclusion:**

Contrary to other studies which did not account for seasonal respiratory viral patterns or concurrent infections, we found an association of concurrent infection with RSV and SARS-CoV-2, but did not find a higher risk of subsequent RSV infection, compared with children seeking care for influenza and other respiratory illnesses during the RSV surge in 2022.

**Disclosures:**

**Asuncion Mejias, MD, PhD, MsCS**, Astra-Zeneca: Advisor/Consultant|Astra-Zeneca: Honoraria|Enanta: Advisor/Consultant|Janssen: Advisor/Consultant|Janssen: Grant/Research Support|Merck: Advisor/Consultant|Merck: Grant/Research Support|Moderna: Advisor/Consultant|Pfizer: Advisor/Consultant|Pfizer: Honoraria|Sanofi-Pasteur: Advisor/Consultant|Sanofi-Pasteur: Honoraria

